# 吹扫捕集-气相色谱-三重四极杆质谱法同时测定饮用出厂水中6种卤乙腈

**DOI:** 10.3724/SP.J.1123.2020.08026

**Published:** 2021-07-08

**Authors:** Wei ZHAN, Zhiyu HAN, Yong LI, Fei LIU, Yong ZHANG

**Affiliations:** 北京市疾病预防控制中心, 北京市预防医学研究中心, 北京 100013; Beijing Center for Disease Prevention and Control, Beijing Research Center for Preventive Medicine, Beijing 100013, China; 北京市疾病预防控制中心, 北京市预防医学研究中心, 北京 100013; Beijing Center for Disease Prevention and Control, Beijing Research Center for Preventive Medicine, Beijing 100013, China; 北京市疾病预防控制中心, 北京市预防医学研究中心, 北京 100013; Beijing Center for Disease Prevention and Control, Beijing Research Center for Preventive Medicine, Beijing 100013, China; 北京市疾病预防控制中心, 北京市预防医学研究中心, 北京 100013; Beijing Center for Disease Prevention and Control, Beijing Research Center for Preventive Medicine, Beijing 100013, China; 北京市疾病预防控制中心, 北京市预防医学研究中心, 北京 100013; Beijing Center for Disease Prevention and Control, Beijing Research Center for Preventive Medicine, Beijing 100013, China

**Keywords:** 气相色谱-三重四极杆质谱, 吹扫捕集, 消毒副产物, 卤乙腈, 出厂水, gas chromatography-triple quadrupole mass spectrometry (GC-MS/MS), purge and trap, disinfection by-products, haloacetonitriles (HANs), finished water

## Abstract

目前卤乙腈作为我国非受监管的消毒副产物广泛存在于饮用出厂水中,可产生多种毒性,缺乏相关标准检测依据。研究建立了吹扫捕集-气相色谱-三重四极杆质谱同时测定饮用出厂水中氯乙腈、二氯乙腈、三氯乙腈、溴乙腈、溴氯乙腈、二溴乙腈的分析方法。吹扫捕集技术应用于卤乙腈的测定,实现了样品经采集后全程自动测定,有害试剂零消耗。同时吹扫捕集法相比固相微萃取法,样品制备的速度更快,成本更低。实验考察了样品6 h内目标组分的稳定性;比较了7#(2,6-二苯基对苯醚)、10#(2,6-二苯基对苯醚/硅胶/碳分子筛)、11#(疏水活性炭)、12#(疏水活性炭)捕集阱对目标组分响应的影响;考察了4种型号色谱柱(VF-5、Rxi-624、DB-VRX、HP-INNOWAX)对色谱峰形的影响。实验条件经优化,确定了吹扫捕集采用10#捕集阱,将25 mL水样于35 ℃吹扫11 min,于190 ℃解析1 min。气相色谱分流进样,分流比1:10,使用Rxi-624Sil MS色谱柱(60 m×0.25 mm×1.40 μm)程序升温分离,线速度30 cm/s,在MRM模式下检测,外标法定量。结果表明,6种卤乙腈的基质效应为0.85~1.09,在各自范围内线性良好,*r*>0.9991,方法检出限为0.8~120.0 ng/L,定量限为1.5~300.0 ng/L, 3水平平均加标回收率为84.2%~106%,相对标准偏差(RSD)为1.81%~10.7%。对38份出厂水样品进行测定,卤乙腈总检出率为92.1%,含量为0.0101~1.28 μg/L。该方法高效、灵敏、环保,为针对卤乙腈类新兴消毒副产物开展监测及健康风险评估提供了优质的技术选择。

## Abstract (en)

Haloacetonitriles (HANs) are widely used in finished water as unregulated disinfection by-products. HANs may pose much threat to human health, and there is no relevant standard examination method for these compounds. A method was established for the simultaneous determination of six HANs (chloroacetonitrile (CAN), dichloroacetonitrile (DCAN), trichloroacetonitrile (TCAN), bromoacetonitrile (BAN), bromochloroacetonitrile (BCAN), and dibromoacetonitrile (DBAN)) in finished water by using purge and trap-gas chromatography-triple quadrupole mass spectrometry (GC-MS/MS). The purge and trap technology helps realize automatic determination of samples after collection, without using any harmful reagent. The cost and analytical efficiency of this method were superior to those of solid phase microextraction (SPME). Considering the instability of HANs, the analysis must be carried out as soon as possible after sampling, in order to avoid significant changes in their concentration during storage. In particular, the use of an appropriate quenching agent was critical to sample collection. In this study, ascorbic acid was chosen as the quenching agent. The stabilities of the spiked samples at the levels of 0.1 (TCAN), 0.2 (CAN), 1.0 (DCAN), 1.0 (BAN), 1.0 (BCAN), 4.0 (DBAN) μg/L were tested. The effect of sample storage time (0, 0.5, 1, 2, 3, 4, and 6 h) on the responses of the target component was evaluated. The stability results showed that within 6 h, the relative standard deviations of the responses for the six HANs ranged from 2.32% to 6.98%. To validate the method, first, different traps, viz. 7# (Tenax), 10# (Teanx/silica gel/cms), 11# (VOCARB), and 12# (BTEXTRAP) were optimized. Second, various chromatographic columns (VF-5, Rxi-624, DB-VRX, and HP-INNOWAX) were compared to investigate their influence on the peak shape. Under the optimal detection conditions, the six HANs in finished water were extracted with the 10# trap. The volume of the water sample was used 25 mL, with purging at 35 ℃ for 11 min, and desorbed at 190 ℃ for 1 min. Chromatographic separation was performed on a Rxi-624Sil MS chromatographic column (60 m×0.25 mm×1.40 μm). Gas chromatographic conditions were obtained under the following conditions: split ratio, 1:10; linear velocity, 30 cm/s. The triple quadrupole mass spectrometer was operated in the electron impact (EI) mode. The target compounds were detected in the multiple reaction monitoring (MRM) mode. Quantitation was carried out using the external standard method. The results showed that the matrix effects of the six HANs ranged from 0.85 to 1.09. Good linearities were obtained in the range of the standard curves. The correlation coefficients (*r*) were greater than 0.9991. The limits of detection (LODs, *S/N*=3) were 0.8-120.0 ng/L. The limits of quantification (LOQs, *S/N*=10) were 1.5-300.0 ng/L. The average recoveries of the six HANs ranged from 84.2% to 106%, and the RSDs were in the range of 1.81%-10.7%. In August 2020, 38 samples of finished water were tested. All of the six HANs were found in the finished water. The concentrations of the HANs were in the range of 0.0101-1.28 μg/L, and the total detection rate was 92.1%. The detection rates of the individual components followed the order DCAN>BCAN>CAN>TCAN>BAN>DBAN. The developed method is efficient, sensitive, and environmentally friendly. It provides a high-quality technical choice for monitoring and health risk assessment of the emerging disinfection by-products of HANs.

饮用水消毒是保障饮水安全的重要手段。卤乙腈(HANs)是含氮类消毒副产物中浓度水平最高的一类,其神经毒性、遗传毒性、细胞毒性已被相继报道^[1,2,3]^;二氯乙腈(DCAN)可引发皮肤癌,三氯乙腈(TCAN)和溴氯乙腈(BCAN)可引发肺癌^[4]^。国际癌研究机构(IARC)分别将二溴乙腈(DBAN)列入ⅡB类,二氯乙腈和三氯乙腈列入Ⅲ类致癌物清单。

卤乙腈广泛存在于饮用出厂水中,液氯、氯胺、二氧化氯、臭氧等多种消毒方式均会导致卤乙腈的产生^[5]^,且因其亲水性的结构使得在水质处理工艺中较难去除^[6]^。卤乙腈较三卤甲烷、卤乙酸毒性更强,且可能产生蓄积效应^[7]^。世界卫生组织(WHO)《饮用水水质准则》(第四版)中规定了二氯乙腈(20 μg/L)和二溴乙腈(70 μg/L)的限值要求^[8]^。日本则对二氯乙腈和二溴乙腈做出了较《饮用水水质准则》更加严格的限值要求^[9]^。但目前该类消毒副产物在我国仍处于非受监管状态,缺乏相关的标准规范。

饮用水样品中卤乙腈的前处理方法有液液萃取法^[10,11]^、液液微萃取法^[12,13,14]^、固相萃取法^[15]^、静态顶空法^[16,17,18]^。但以上方法均存在不足之处,其中液液萃取法与固相萃取法均需消耗有害试剂;液液微萃取法步骤繁琐且难以实现自动化连续进样;静态顶空法灵敏度较低。寻求应用高自动化、环境友好的样品前处理技术是目前的研究趋势。因此近年来固相微萃取法被相继报道^[19,20,21,22]^。与固相微萃取法相比,吹扫捕集法同为绿色化学技术,且普及性更强,样品制备的速度更快,成本更低。但目前将吹扫捕集法应用于卤乙腈检测的相关报道较少。仪器方法选择方面,现阶段的文献报道有气相色谱法(GC)^[17,23,24]^、气相色谱-质谱法(GC-MS)^[11-14,16,18-21]^、气相色谱-串联质谱法(GC-MS/MS)^[15,25]^、柱后荧光衍生液相色谱法^[26]^。GC可使用氢离子火焰检测器(FID)^[23]^、电子捕获检测器(ECD)^[10,17]^、卤素特殊检测器(XSD)^[24]^检测卤乙腈。其中ECD和XSD较四极杆质谱检测器具有更高的灵敏度。但GC仅凭保留时间定性分析,检测特异性不足,易受干扰。综合评判,GC-MS/MS可兼具特异性检测和灵敏度的优势。

本研究采用吹扫捕集-气相色谱-三重四极杆质谱法同时测定饮用出厂水中最典型的6种卤乙腈(氯乙腈(CAN)、二氯乙腈、三氯乙腈、溴乙腈(BAN)、溴氯乙腈、二溴乙腈)。配合优化后的25 mL吹扫捕集样品制备系统,实现样品经采集后全程自动测定,有害试剂零消耗。在对38份出厂水样品的检测中,6种目标组分均有检出,方法表现出优越的适用性,应用前景广阔。

## 1 实验部分

### 1.1 仪器、试剂与材料

GC-MS-TQ 8040气相色谱-质谱仪(日本Shimadzu公司)、Eclipse 4660吹扫捕集仪(美国OI Analytical公司)、Milli-Q超纯水仪(美国Millipore公司)、XPE-105电子天平(瑞士Mettler-Toledo公司)。

二氯乙腈、三氯乙腈、溴氯乙腈、二溴乙腈标准溶液(5.0 mg/mL)均购自美国AccuStandard公司;氯乙腈(纯度>98%)、溴乙腈(纯度>97%)均购自日本TCI公司;抗坏血酸(分析纯,北京化学试剂公司)。

样品采自本地市、区两级所辖自来水厂。

### 1.2 标准溶液的配制

准确称取氯乙腈、溴乙腈标准品0.0100 g,以丙酮稀释定容至10 mL,配制1000 mg/L的氯乙腈、溴乙腈单标准储备液。分别准确移取氯乙腈和溴乙腈储备液0.05 mL和0.25 mL,以丙酮稀释定容至100 mL,即得2种卤乙腈混合标准使用液。准确移取混合标准使用液5、10、20、40、80、100 μL,以超纯水定容至50 mL,获得系列标准溶液后,立即测定。

分别准确移取5.0 mg/mL的三氯乙腈、二氯乙腈、溴氯乙腈、二溴乙腈储备液5、50、50、200 μL,以丙酮稀释定容至100 mL,即得4种卤乙腈混合标准使用液。准确移取混合标准使用液2、5、10、20、40、80 μL,以超纯水定容至50 mL,获得系列标准溶液后,立即测定。

### 1.3 样品的采集

样品采集时应尽可能充满顶空瓶。对于出厂水样品,每40 mL样品加入30 mg抗坏血酸去除残留消毒剂干扰。样品采集后可直接上机测定,并于6 h内完成。

### 1.4 仪器分析条件

1.4.1 吹扫捕集条件

捕集阱:10#(含有2,6-二苯基对苯醚/硅胶/碳分子筛),吹扫体积:25 mL,吹扫温度:35 ℃,吹扫时间:11 min,解吸温度:190 ℃,解吸时间:1 min,吹扫流量:40 mL/min。

1.4.2 色谱-质谱条件

色谱柱:Rxi-624Sil MS柱(60 m×0.25 mm×1.40 μm),进样口温度:220 ℃,载气:高纯氦气,线速度:30 cm/s,分流比:1:10。升温程序:初温40 ℃,保持5 min,以6 ℃/min升温至110 ℃,保持4 min,以10 ℃/min升温至190 ℃,再以20 ℃/min升温至220 ℃,保持5 min。

离子源:电子轰击电离(EI)源;离子源温度:230 ℃,接口温度:230 ℃,扫描间隔0.3 s,检测器电压:调谐电压+0.6 kV,扫描模式:多反应监测(MRM)模式,溶剂延迟:12 min。6种卤乙腈的保留时间和质谱参数见表1。

**表 1:** 6种卤乙腈的保留时间和质谱参数

Compound	Retention time/min	CAS No.	Quantitative ion pair (m/z)	Qualitative ion pair (m/z)	Collision energies/V
Trichloroacetonitrile (TCAN)	14.516	545-06-2	108.0>73.0	108.0>47.0	25, 20
Chloroacetonitrile (CAN)	17.112	107-14-2	75.0>48.0	75.0>40.1	8, 15
Dichloroacetonitrile (DCAN)	18.829	3018-12-0	74.0>47.0	82.0>47.0	15, 25
Bromoacetonitrile (BAN)	21.565	590-17-0	119.0>40.1	121.0>40.1	10, 10
Bromochloroacetonitrile (BCAN)	23.529	83463-62-1	74.0>47.0	154.9>74.0	15, 8
Dibromoacetonitrile (DBAN)	27.102	3252-43-5	199.0>117.9	120.0>92.9	8, 25

## 2 结果与讨论

### 2.1 吹扫捕集条件的优化

2.1.1 捕集阱型号的选择

捕集阱是影响目标组分检测的核心因素之一。采用控制单一变量的方法,分别考察7#(2,6-二苯基对苯醚)、10#(2,6-二苯基对苯醚/硅胶/碳分子筛)、11#(疏水活性炭)、12#(疏水活性炭)捕集阱对典型样品(均未检出二溴乙腈)的测定结果。以10#捕集阱响应值为分母,与各型号捕集阱响应值做比,比值结果见图1。其中,11#捕集阱不适宜溴乙腈、溴氯乙腈的检测,12#捕集阱不适宜三氯乙腈、溴乙腈、溴氯乙腈的检测。7#捕集阱虽然对氯乙腈和二氯乙腈的捕集效果更佳,但综合考虑氯乙腈和二氯乙腈的灵敏度均较高,优先选择更有利溴乙腈和溴氯乙腈检测的10#捕集阱。

**图 1:**
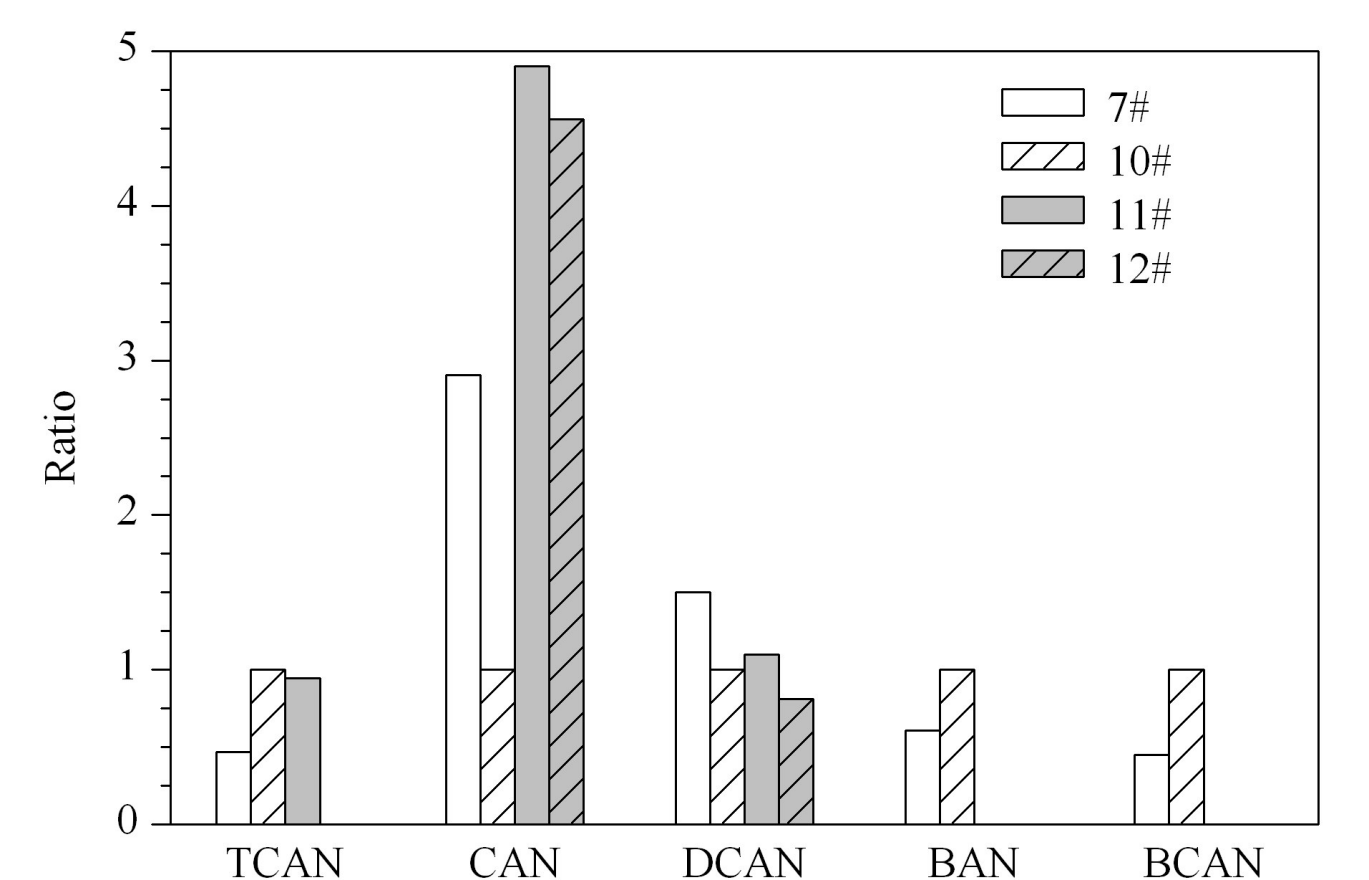
4种型号捕集阱对典型样品测定的影响

2.1.2 解吸时间与解吸温度的选择

本研究采用10#捕集阱,其产品手册明确给出推荐的解吸温度为190 ℃,推荐解吸时间为0.5~4.0 min。故确定解吸温度为190 ℃。实验对比1、2、3、4 min的解吸时间对目标组分响应的影响。结果表明,随解吸时间的增加,6种卤乙腈的响应值无上升趋势,其峰面积相对标准偏差均小于3.1%。因此确定解吸时间为1 min。

### 2.2 气相色谱-质谱条件的优化

2.2.1 气相色谱条件的选择

色谱柱是影响目标组分峰形的关键因素,尖锐对称的色谱峰有利于准确积分,提高定量准确性。实验分别考察弱极性的VF-5柱(60 m×0.25 mm×0.25 μm),中等极性DB-VRX柱(60 m×0.25 mm×1.40 μm)、Rxi-624柱(60 m×0.25 mm×1.40 μm),极性HP-INNOWAX柱(30 m×0.25 mm×0.25 μm)对卤乙腈检测的影响。VF-5柱对6种卤乙腈普遍存在拖尾现象。DB-VRX柱对氯乙腈、溴乙腈存在拖尾现象,响应较弱。Rxi-624柱和HP-INNOWAX柱均对6种卤乙腈检测效果良好,HP-INNOWAX柱对改善氯乙腈和溴乙腈的峰形更为有利,但鉴于其使用上限温度较低,综合柱流失等因素,故本研究选择Rxi-624色谱柱。6种卤乙腈的总离子流色谱图见图2。

**图 2:**
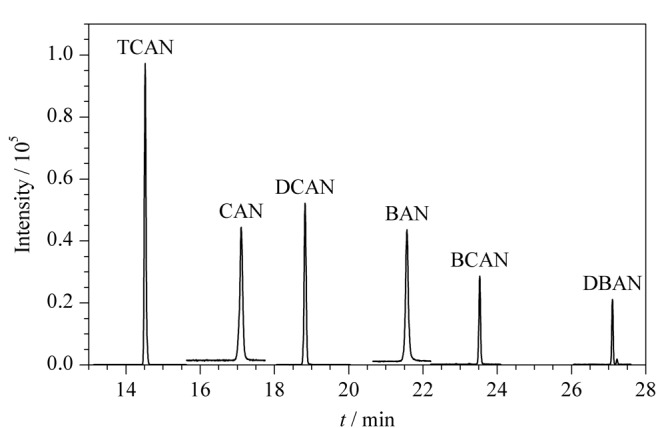
6种卤乙腈的总离子流色谱图

2.2.2 质谱参数的选择

实验首先进行全扫描,查找目标组分,确定一级质谱信息。其次进行产物离子扫描,前体离子经诱导碰撞,获得二级质谱信息。通常选择一级质谱信息中的基峰作为前体离子。以二溴乙腈为例,分别以基峰(*m/z*=120.0)和*m/z*为199.0的碎片离子为前体离子,对比二级质谱信息发现,虽然*m/z*为199.0的前体离子的强度只有基峰的40%左右,但能够获得更强的二级质谱信息。确定二溴乙腈定量离子对*m/z*为199.0>117.9,定性离子对*m/z*为120.0>92.9。

2.2.3 SIM与MRM采集模式的比较

鉴于GC-MS/MS设备较为昂贵,普及程度尚不如GC-MS。本研究同时应用SIM模式对加标样品进行测定,并与MRM模式进行比较(见图3)。MRM在检测灵敏度、特异性和基线状态等方面具有优势。

**图 3:**
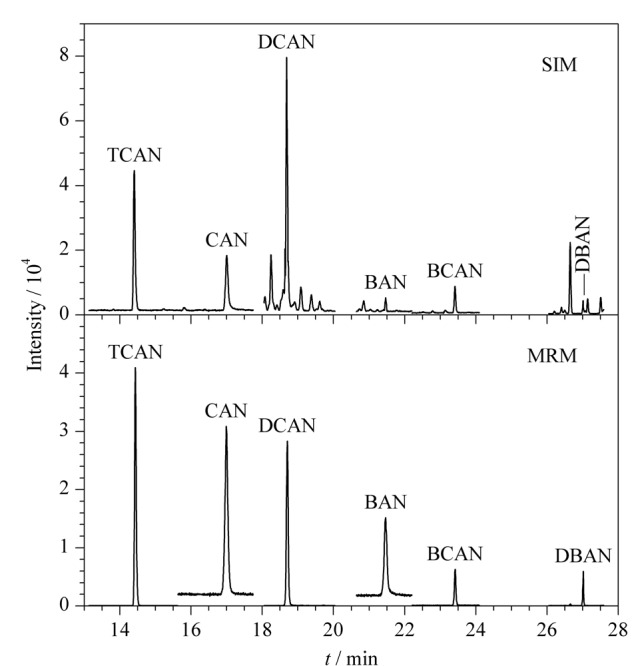
SIM和MRM模式下加标样品中6种卤乙腈的总离子流色谱图

### 2.3 基质效应评价

基质效应=基质匹配校准曲线的斜率/溶剂校准曲线的斜率,比值越接近1,则基质效应越小;若比值为0.8~1.2,则表明基质效应不明显^[27]^。实验分别以阴性出厂水样品和超纯水建立相应的标准曲线,经计算,6种卤乙腈的基质效应为0.85~1.09。故本研究采用外标法定量,以超纯水为溶剂配制系列标准溶液。

### 2.4 样品采集和稳定性

针对含有消毒副产物样品饮用水的采集需选择适宜的淬灭剂终止残留消毒剂的影响。参考Kristiana等^[28]^研究结果,本研究选择抗坏血酸作为淬灭剂。

实验过程发现,卤乙腈在水体系中稳定性较差,主要表现为三氯乙腈的衰减;二溴乙腈衰减并能产生溴乙腈;溴氯乙腈衰减并能产生氯乙腈。因此样品采集后应尽快测定,避免定量分析时各组分间可能的相互干扰。配制加标样品,质量浓度为0.1(三氯乙腈)、0.2(氯乙腈)、1.0(二氯乙腈)、1.0(溴乙腈)、1.0(溴氯乙腈)、4.0 (二溴乙腈) μg/L,分别于0、0.5、1、2、3、4、6 h进行测定。结果显示,6种卤乙腈峰面积的RSD为2.32%~6.98%,样品采集后6 h内完成测定可保证结果的准确性。

### 2.5 方法学评价

2.5.1 标准曲线、检出限与定量限

以各组分浓度为横坐标(*X*, μg/L)、定量离子峰面积为纵坐标(*Y*)绘制标准曲线,相关系数(*r*)≥0.9991。以定量离子的信噪比(*S/N*)为3和10时的浓度分别确定方法的检出限和定量限,分别为0.8~120.0 ng/L和1.5~300.0 ng/L(见表2)。

**表 2:** 6种卤乙腈的线性范围、线性方程、相关系数、检出限和定量限

Compound	Linear range/(μg/L)	Linear equation	r	LOD/(ng/L)	LOQ/(ng/L)
TCAN	0.01-0.4	Y=1.23×10^6^X-1.75×10^3^	0.9996	0.8	1.5
CAN	0.05-1.0	Y=2.03×10^5^X+2.10×10^3^	0.9999	10.0	25.0
DCAN	0.10-4.0	Y=7.37×10^4^X-4.55×10^3^	0.9996	8.0	20.0
BAN	0.25-5.0	Y=1.16×10^4^X+1.88×10^3^	0.9991	100.0	200.0
BCAN	0.10-4.0	Y=3.49×10^4^X+8.58×10^2^	0.9998	20.0	50.0
DBAN	0.40-16	Y=2.45×10^3^X+6.38×10^2^	0.9999	120.0	300.0

*Y*: peak area; *X*: mass concentration, μg/L.

2.5.2 回收率与精密度

以空白饮用出厂水为样品,添加低、中、高3个水平的混合标准溶液,进行加标回收及精密度试验(*n*=5)。6种卤乙腈的回收率为84.2%~106%,相对标准偏差为1.81%~10.7%(见表3)。

**表 3:** 6种卤乙腈的平均回收率和相对标准偏差(*n*=5)

Compound	Spiked levels/ (μg/L)	Recoveries/%	RSDs/%
TCAN	0.05, 0.1, 0.30	95.3, 88.0, 84.2	7.86, 9.90, 7.67
CAN	0.1, 0.2, 0.60	98.8, 100, 99.5	7.08, 6.11, 5.33
DCAN	0.5, 1, 3.0	97.0, 104, 102	4.44, 6.24, 1.81
BAN	0.5, 1, 3.0	94.6, 91.8, 94.6	5.76, 4.05, 8.44
BCAN	0.5, 1, 3.0	97.5, 105, 96.8	7.24, 7.22, 4.23
DBAN	2, 4, 12	104, 106, 103	9.69, 4.30, 10.7

2.5.3 实际样品测定

应用本研究建立的方法,于2020年8月对本市38份市政饮用出厂水开展检测,6种卤乙腈均有检出。单份样品中若以其中任意卤乙腈的检出为标准,样品总检出率为92.1%。各组分检出率依次为二氯乙腈>溴氯乙腈>氯乙腈>三氯乙腈>溴乙腈>二溴乙腈,具体样品测定结果见表4。卤乙腈检出含量远低于WHO中二氯乙腈(20 μg/L)和二溴乙腈(70 μg/L)的限值要求^[8]^。

**表 4:** 38份出厂水中6种卤乙腈的检测结果

Compound	Number of positive samples	Detection rate/%	Content/ (μg/L)	
TCAN	22	57.9	0.0101-	0.0484
CAN	31	81.6	0.0576-	0.104
DCAN	35	92.1	0.115-	1.28
BAN	12	31.6	0.292-	0.392
BCAN	34	89.5	0.114-	0.445
DBAN	10	26.3	0.418-	0.484

## 3 结论

本研究采用吹扫捕集-气相色谱-三重四极杆质谱法对饮用出厂水中6种典型卤乙腈开展检测。方法符合绿色化学理念,满足大批量检测要求,高效便捷,具有较高的推广价值。同时该项技术可与常见挥发性有机物联合检测,为饮用水监测领域开发高通量的实用性方法奠定基础。
